# Chlorogenic acid against palmitic acid in endoplasmic reticulum stress-mediated apoptosis resulting in protective effect of primary rat hepatocytes

**DOI:** 10.1186/s12944-018-0916-0

**Published:** 2018-11-28

**Authors:** Yong Zhang, Liangsheng Miao, Huijuan Zhang, Gang Wu, Zhenni Zhang, Jianrui Lv

**Affiliations:** 1 Department of Anesthesiology, Second Affiliated Hospital of Xi’an Jiaotong University, No. 157, West 5th Road, Xi’an, 710004 Shaanxi Province China; 2 Department of Anesthesiology, Weinan Central Hospital, No.7, East of Chaoyang Road, Weinan, 714000 Shaanxi Province China

**Keywords:** Palmitic acid, Chlorogenic acid, ER stress, Hepatocytes, Liver

## Abstract

**Background:**

We demonstrated growing evidence supports a protective role of chlorogenic acid of rat hepatocytes elicited by two compounds, i.e. thapsigargin and palmitic acid. Nevertheless, little is known about the mechanisms of palmitic acid induced endoplasmic reticulum (ER) stress and cell death.

**Methods:**

The proliferation of primary rat hepatocytes was detected by MTT assay. The expression of GRP78, CHOP and GRP94 was detected by Western blot analyses.

Caspase-3 activity was detected by a Caspase-3 substrate kit. Cell apoptosis was detected by Hoechst 33342 staining.

**Results:**

We demonstrated that incubation of hepatocytes for 16 h with palmitic acid elevated cell death. Moreover, Western blot analyses demonstrated increased levels of the endoplasmic reticulum stress markers — glucose regulated protein 78 (GRP78), C/EBP homologous protein (CHOP), and glucose regulated protein 94 (GRP94). Chlorogenic acid could inhibit ER stress induced cell death and levels of indicators of ER stress caused by palmitic acid. The effect of thapsigargin, which evokes ER stress were reversed by chlorogenic acid.

**Conclusions:**

Altogether, our data indicate that in primary rat hepatocytes, chlorogenic acid prevents ER stress-mediated apoptosis of palmitic acid.

## Introduction

Hepatocyte death is associated with almost every hepatopathy.

In recent studies, thapsigargin (TG) has found such widespread use since it pumps Ca^2+^ from the cytosol into the lumen of the endoplasmic reticulum (ER) in cells [1].

During the last thirty years, the mechanism of TG action has been illustrated thoroughly [2]. As we all know that raising of intracellular free calcium([Ca^2+^]i) may cause cell death in many cells such as hepatocytes [3].

Saturated fatty acids (FA) including palmitic acid may cause apoptosis and ER stress in rat and human liver cell lines [4–8], which will result in degeneration and/or inflammation.

The assumption is confirmed by the fact that palmitic acid induced ER stress and apoptosis are founded in mice and rats [9, 10].

Mechanisms of hepatotoxicity are complex and one drug may have several toxic mechanisms occurring at the same time or sequentially.

Coffee has higher concentration of polyphenols among the beverages [11] . Chlorogenic acid is the major polyphenol in coffee. There are a large number of phenolics exited in promotive health foods and in the plant kingdom, such as vegetables and fruits. Phenolics are also commonly found in beverages made from plants, such as tea, coffee, and wine [12]. In numerous biological tests, Chlorogenic acid has been proved to have superoxide anion-scavenging effects, in other words, it has the ability to suppress hepatitis B virus and to restrain lipid peroxidation. According to the study, the restriction of ER stress may cause the protective effect of chlorogenic acid in CCl_4_-treated rats [13]. Chlorogenic acid induce apoptosis in several types of cancer cells in vitro [14–16]. However, until now, we have very limited information to prove the toxicity of polyphenols and related phenolics to normal cells. According to recent studies, chlorogenic acid is likely to reduce the risk of oxidative cell death [17–19].

In the present study, whether the chlorogenic acid could protect rat primary hepatocytes and conduce to clinically-relevant ER stress inducer such as palmitic acid was examined. In this paper, we report that: (1) palmitic acid could induce ER stress and apoptosis in hepatocytes; (2) chlorogenic acid could reduce cell death induced by palmitic acid; (3) with specific attention given to GRP78, GRP94 and CHOP, we can adjust the effects through alteration of the ER stress.

## Materials and methods

### Materials and cells

Hepatocytes were prepared as previously described [20]. The cells were plated on 35-mm diameter culture plates (1 × 10^6^ cells/plate) in M199 containing 1% (*v*/v) antibiotics (10 U/μg penicillin, 10 μg/μL streptomycin), 100 nM dexamethasone, 0.5 nM insulin and 4% (*v*/v) NCS. After initial 4 h incubation for the attachment of the cells to the substratum, the medium was changed to Williams’ medium E 1% (v/v) antibiotics (10 U/μg penicillin, 10 μg/μL streptomycin), 100 nM dexamethasone and 0.5 nM insulin without NCS. Experimental treatments were performed after 44 h of culture in Williams’ medium E containing 1% (v/v) antibiotics and 100 nM dexamethasone.

### Incubation of hepatocytes

The cells at 85–95% confluence, were incubated with palmitic acid (250 μmol/l) or thapsigargin (5 μmol/l) for up to 16 h. Then hepatocytes were incubated with palmitic acid (250 μmol/l) with/without chlorogenic acid (1 or 5 μmol/l) for up to 16 h.

### Measurement of cell viability and death

Cell death was assessed by measurement of lactate dehydrogenase (LDH) from lysed cells, Annexin V Fluorescein (FITC) and propidium iodide (PI) double staining assay (BD Biosciences, USA) was used to quantify apoptosis rates using FACScan flow cytometer (Becton-Dickinson, USA). After treatment of morphine and oxycodone alone or together with nalmefene for 48 h in 6-well plates, cells were collected and wash with cold PBS. After centrifugation with 1000r/3 min, cells were re-suspended using 100 μl binding buffer with 1 × 10^5^ cells each group. Then Annexin V and PI were added according to instruction of test kits. After incubated for 15 mins at room temperature in the dark, samples were analyzed by flow cytometry. Cells without any treatment were used as negative control. Data was attained by analyzing early apoptotic rates (Annexin V+/PI-) and late apoptotic rates (Annexin V+/PI+).

### Measurement of caspase-3 activation

Caspase-3 activity was evaluated using a DEVD-NucViewTM 488 Caspase-3 substrate kit (Biotium Inc., Cambridge, UK). In the presence of active caspase-3 enzyme, the substrate dissociates from its bound fluorogenic DNA-binding dye and the latter binds to DNA and emits fluorescence. Caspase-3 was detected by microscopic examination and also by adapting the kit for microplate fluorescence reading. For this, cells were incubated with 50 μL of 5 μmol/L DEVD-NucView™ 488 Caspase-3 substrate for 30 min. Fluorescence was measured in a microplate reader (Cary Eclipse, Varian Inc.) set at wavelengths of 490 nm excitation and 520 nm emission.

### Western blot analysis

Cells were washed in ice-cold PBS twice, and lysed in buffer with protease inhibitor, and phosphatase inhibitor, and then centrifuged at 13000×g for 25 min at 4 °C. The supernatant was collected and total proteins were quantified using bicinchoninic acid (Pierce, Rockford, AL, USA,) method. The protein samples were loaded onto polyacrylamide gel and subjected to sodium dodecyl sulfate polyacrylamide gel electrophoresis (SDS-PAGE). Proteins were then transferred onto a polyvinylidenedifluoride (PVDF) membrane. The membrane was blocked with Tris-buffered saline and Tween 20 (TBST) containing 4% BSA for 1 h at room temperature. The membranes were incubated serially with primary antibodies at 4 °Covernight. After washing with TBST 3 times for 8 min each, the membranes were incubated with secondary antibodies for 1 ± 2 h at room temperature. The density of the corresponding bands was measured quantitatively using image analysis software (Bio-Red, Hercules, CA, USA) and corrected by reference to the value of β-actin.

### Statistical analysis

All results were reported as mean ± SD from three independent experiments. Cell survival, proliferation and differentiation among different groups were compared using SPSS statistical software (version 12.0). Statistical significance was determined using Student’s t-test. *P* < 0.05 was considered statistically significant.

## Results

### Chlorogenic acid inhibited palmitic acid induced cell death

**Fig. 1:**
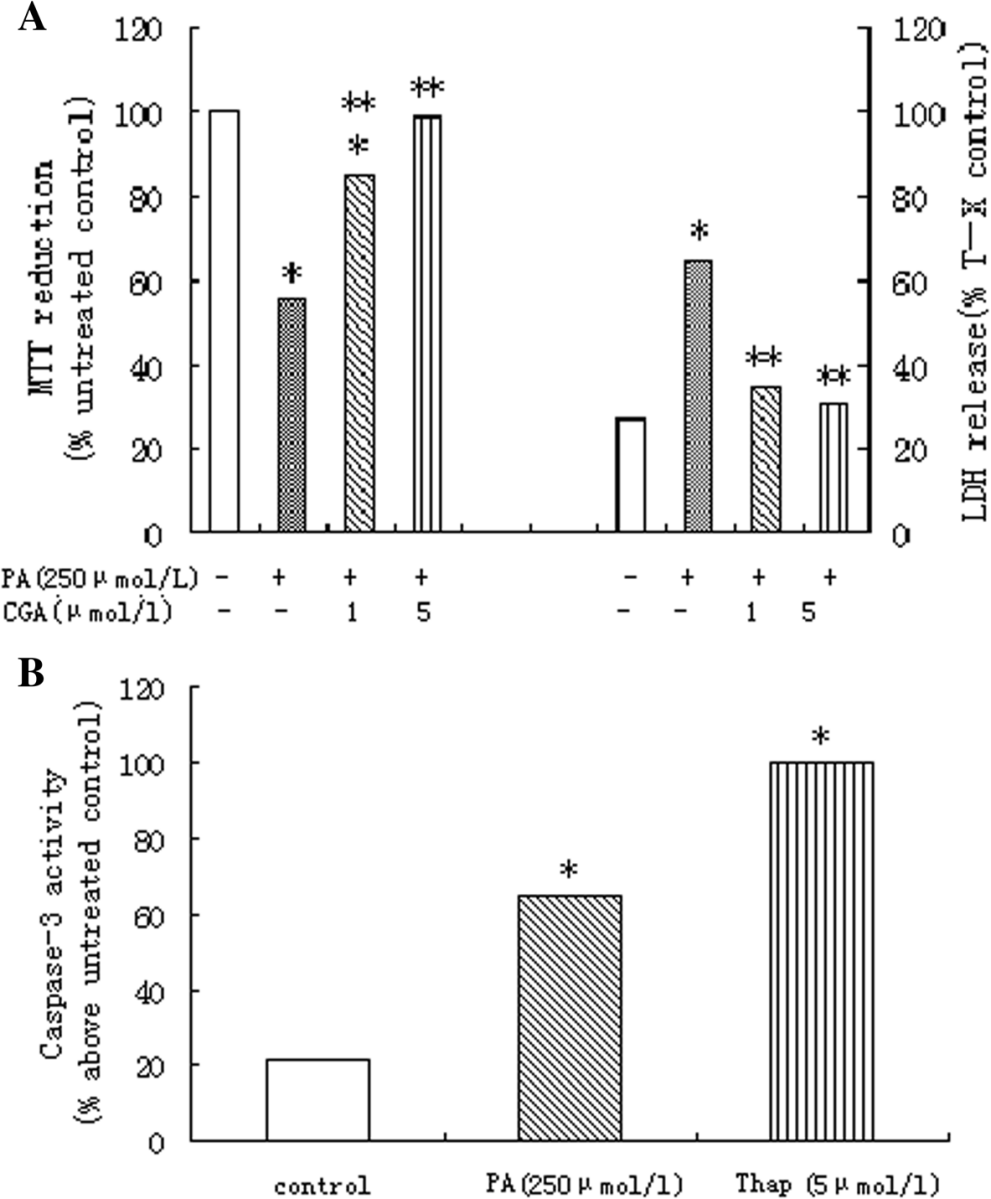
**a** Primary rat hepatocytes treated with 250 μmol/l palmitic and 5 μmol/l chlorogenic acid presented restored cell viability to levels observed in untreated cells on MTT reduction and LDH release. Data represent mean ± S.E.M., *n* = 5, *P<0.05 vs. control (0 μmol/l palmitic acid), **P<0.05 vs. palmitic acid-only cells. **b** Treatment of primary rat hepatocytes with 250 μmol/L palmitic acid produced a significant increase in activity of caspase-3. For comparison, the effects of thapsigargin(Thap; 5μmol/l)are also shown. Data represent mean±S.E.M., n=5, *P< 0.05 vs. control (0 μmol/l palmitic acid), **P< 0.05 vs. palmitic acid-only cells.

Primary rat hepatocytes treated with 250 μmol/l palmitic and 5 μmol/l chlorogenic acid presented restored cell viability to levels observed in untreated cells (Fig. 1).

Compared with 5 μmol/l chlorogenic acid, 1 μmol/l chlorogenic acid showed weaker restored cell viability effects on primary rat hepatocytes.

### Chlorogenic acid reduces ER stress mediated by palmitic acid

**Fig. 2:**
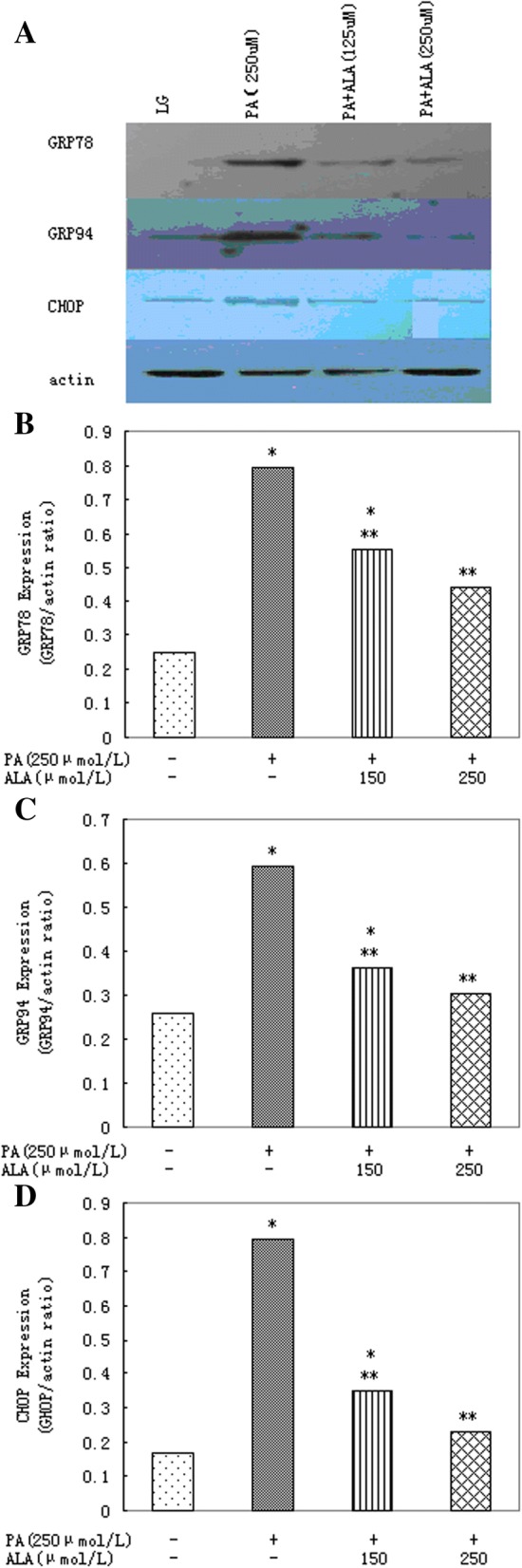
Chlorogenic acid protects primary rat hepatocytes against ER stress induced by palmitic acid. **a** Western blot and (**b**) densitometric analysis demonstrating the reduction of palmitic acid (PA)-induced GRP78 expression by 1 or 5 μmol/l chlorogenic acid (CGA) after 16 h. **a** Western blot and (**c**) densitometric analysis of GRP94 expression after 16 h incubation of cells with 250 μmol/l palmitic acid (PA) in presence of 1 or 5 μmol/l chlorogenic acid (CGA). **a** Western blot and (**d**) densitometric analysis demonstrating the reduction of palmitic acid (PA)-induced CHOP expression by 1 or 5 μmol/l chlorogenic acid (CGA) after 16 h. Data represent mean ± S.E.M., *n* = 5, *P<0.05 vs. LG, low glucose control (0 μmol/l palmitic acid), **P<0.05 vs. palmitic acid only cells

The immunoblot analysis revealed the presence of GRP78, GRP94 and CHOP After 16 h incubation with palmitic acid (Fig. 2).

We observed exposure of these cells to palmitic acid promoted up-regulation of ER stress markers. Co-incubation 1 or 5 μmol/l chlorogenic acid reduced the levels of GRP78, GRP94 and CHOP after 16 h (Fig. 2).

### Chlorogenic acid reduced death caused by thapsigargin

**Fig. 3:**
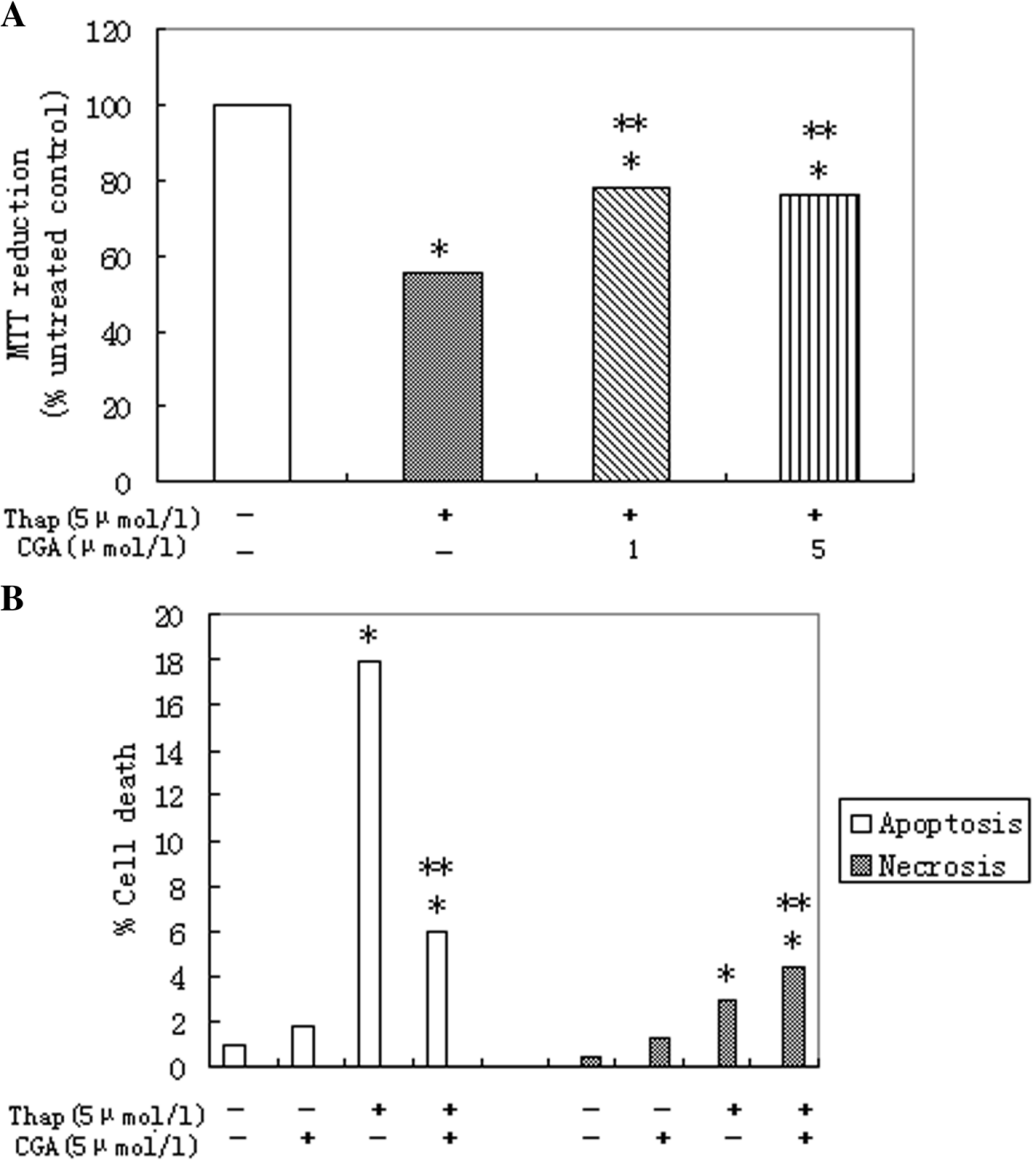
Chlorogenic acid protects against dysfunction and apoptosis of primary rat hepatocytes induced by thapsigargin. **a** MTT reduction. **b** Cell death. Relative cell death treated with 5 μmol/l thapsigargin (Thap) for 16 h in presence of 1 or 5 μmol/l chlorogenic acid (CGA). Data represent mean ± S.E.M., *n* = 5, *P<0.05 vs. LG, low glucose control set to 1 (0 μmol/l thapsigargin), **P<0.05 vs. thapsigargin-only cells

5 μmol/l thapsigargin causes severe cell death (Fig. 3a). Eighter 1 or 5 μmol/l chlorogenic acid can significantly enhance cell vitality (Fig. 3a) which was confirmed by significant increases in both apoptosis and necrosis (Fig. 3b). 5 μmol/l chlorogenic acid increased cell death mediated by thapsigargin (Fig. 3b).

### Effects of chlorogenic acid on ER stress induced by thapsigargin

**Fig. 4:**
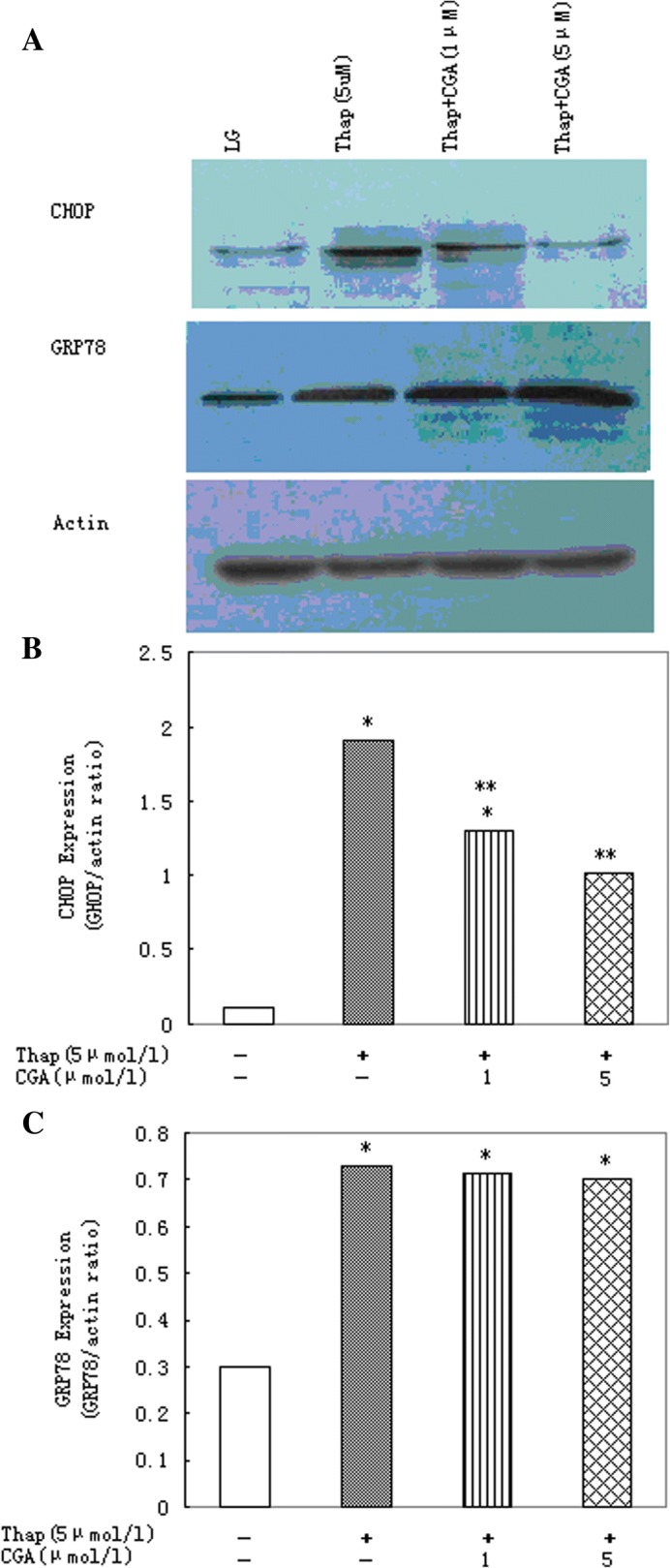
Chlorogenic acid (CGA) protects primary rat hepatocytes against ER stress induced by thapsigargin. **a** Western blot image and densitometric analysis of (**b**) CHOP and (**c**) GRP78 expression in cells treated with thapsigargin (Thap; 5 μmol/l) in presence of increasing concentrations of chlorogenic acid (CGA) for 16 h. Data represent mean ± S.E.M., *n* = 5, *P<0.05 vs. LG, low glucose control (0 μmol/l thapsigargin), **P<0.05 vs. thapsigargin-only cells

5 μmol/l chlorogenic acid decreased the elevated CHOP levels caused by 5 μmol/l thapsigargin (Fig. 4a, c). Neither 5 μmol/l nor 1 μmol/l concentration of chlorogenic acid could reduce GRP78 expression mediated by thapsigargin (Fig. 4a, b).

## Discussion

The objectives of this study were to find out whether chlorogenic acid could prevent palmitic acid induced ER stress and the cell death in liver cells. It has been reported in many cell lines that apoptosis via endoplasmic reticulum (ER) stress. ER stress plays a significant role in many liver diseases.

We go into the effects of chlorogenic acid and palmitic acid, and their combined action about resulting in apoptosis, cell death, ER stress and caspase-3 activity in hepatocytes. We detected the three ER stress markers.

This phenomenon, called lipotoxicity, has been related to cardiac failure, NAFLD and diabetes [21–24] . UPR activation was founded in NAFLD, cardiac dysfunction and hyperadiposis [25, 26] .

Activation the UPR caused ER stress and cell death, which is bring out by an excess of saturated fatty acids in many cell types [4, 27–29]. Palmitic acid activates ER stress and has been suggested to play a crucial role in the NAFLD. Therefore, damages in ER stabilization is the cause of many diseases and contributes to lipotoxicity. The purpose of our study is to find out the relationship between the saturated fatty acids and ER stress. Our study indicates that (1) chlorogenic acid can reduce cellular dysfunction and apoptosis caused by thapsigargin. (2) chlorogenic acid can reduce cellular dysfunction and apoptosis caused by palmitic acid. (3) by reducing ER stress and apoptosis, chlorogenic acid protects hepatocytes from palmitic acid’s lipotoxicity.

Thapsigargin gives rise to necrosis and apoptosis in many cells [30–32]. We are not clear about how the thapsigargin works to give rise to necrosis and apoptosis. It is possible that chlorogenic acid reduced damages of primary rat hepatocytes by protecting cells against thapsigargin-induced apoptosis.

A number of recent studies have pointed out that many ER stress markers were active during saturated fatty acid-induced apoptosis [4, 33, 34].

Chlorogenic acid has been extensively studied since it is widely distributed in plants, which is one of the main polyphenols in the human diet, and it possesses many health-promoting properties. So, it has the potential to become a health product such as functional food and nutraceuticals.

Functional food and nutraceuticals have the potential to become the future of primary prevention in dyslipidaemia treatment in many diseases [35]. Because of the limited production conditions, it has not been popularized in the market.

Chlorogenic acid has multifunctional properties as a nutraceutical and food additive. As a nutraceutical, chlorogenic acid has anti-oxidant, anti-inflammatory, anti-obesity, antidyslipidemia, antidiabetic, and antihypertensive properties, which can serve for the prevention and treatment of metabolic syndrome and associated disorders [36].

Our follow-up work will focus on animal experiments, which will be done to prove that the in vitro results can be reproduced in the animals. And lack of preclinical tox studies of cholorgenic acid is also a deficiency of our research work. We will make up for it in the work of future research.

## Conclusions

Chlorogenic acid, the major polyphenol in coffee can reduce ER stress produced by palmitic acid. Chlorogenic acid afford protective effect by reduce ER stress. Caspase-3 and CHOP was associated with lipotoxicity of palmitic acid.

Polyphenol such as chlorogenic acid may protect hepatocytes against palmitic acid.

## Abbreviations

CHOP: C/EBP homologous protein
ER: Endoplasmic reticulum
GRP78: Glucose regulated protein 78
GRP94: Glucose regulated protein 94
LDH: Lactate dehydrogenase
